# Biomechanical study of rotational micromovement of the pedicle screw

**DOI:** 10.1186/s40064-016-2694-3

**Published:** 2016-07-08

**Authors:** Tetsutaro Mizuno, Yuichi Kasai, Toshihiko Sakakibara, Takamasa Yoshikawa, Tadashi Inaba

**Affiliations:** Department of Spinal Surgery and Medical Engineering, Mie University Graduate School of Medicine, 2-174 Edobashi, Tsu City, Mie 514-8507 Japan; Department of Mechanical Engineering, Mie University, 1577 Kurimamachiyacyo, Tsu City, Mie 514-8507 Japan

**Keywords:** Lumbar spine, Spinal instrumentation, Biomechanics, Pedicle screw fixation, Loosening, Instrumentation failure

## Abstract

**Background:**

In regard to the fixation using a pedicle screw (PS) and rod system, the mechanism from the onset of the clear zone up to the development of loosening of the pedicle screw is not completely clarified. The purpose of this study is to determine the cause of the pedicle screw loosening by performing a biomechanical study with three-dimensional movie analysis.

**Methods:**

Ten PS fixation model of the lumbar spines (L3–4) of boar cadavers were used. The rotational angles of the L3 and L4 vertebral body and the screw at the time of applying a ±5 Nm load in the left anterior and right posterior flexion directions respectively were calculated based on those at the time of applying no load. The absolute value of the difference in the rotational angles between each vertebral body with left anterior flexion and right posterior flexion and the inserted screws was defined as rotational micromovement.

**Results:**

In both the left anterior and right posterior flexion directions, there were significant differences (p < 0.05) in the rotational angles between the screw and the vertebral body for both the L3 and L4 vertebral bodies.

**Conclusion:**

Our biomechanical results showed that rotational micromovement occurred between the PS and the vertebral body, and repeated rotational micromovement might cause loosening of the screw or pullout of PS fixation.

## Background

Loosening of the screw or pullout of PS fixation occurs in some patients postoperatively (Aghayev et al. 2014; Sandén et al. 2004; Schatzker et al. 1975). The biomechanical mechanism of development of loosening of the pedicle screw is little known (Galbusera et al. 2015; Tokuhashi et al. 2008). We have investigated the biomechanical study and produced the results of weakness of PS fixation under rotational stress, and it may be the cause of loosening of the screw. Thus, in order to study the biomechanical cause of the pedicle screw loosening, a study with three-dimensional movie analysis using a functional spinal unit (hereinafter, FSU) of the lumbar spine of cadaver boars was performed.

## Methods

**Fig. 1:**
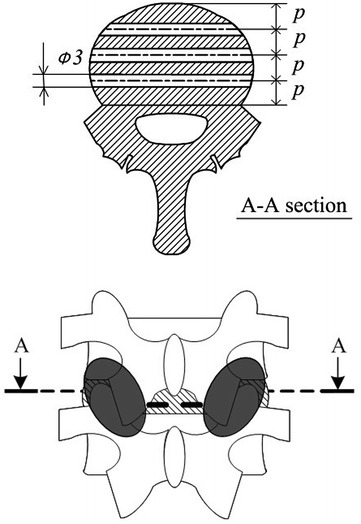
Damaged functional spinal unit

This study was conducted using ten lumbar spine FSUs (L3/4) extirpated from the cadavers of Japanese boars that were captured for Wild Animals Damage Prevention and eventually used for food. The age of boars are approximately 2–3 years old and the average size of boar vertebral body was 17 mm antero-posterior diameter, 25 mm transverse diameter, 25 mm height. The boar pedicle is elliptical shape with 20 mm major axis and 7 mm minor axis. The lumbar spines that were cryogenically-preserved at −30 °C were spontaneously unfrozen, and unnecessary muscles and fat other than internal stability elements were removed. Both ends were attached with dental resin and fixated on a jig. As a model, the specimens were prepared as follow: 3-mm-diameter holes were drilled at 3 locations 1/4, 1/2, and 3/4 from the anterior part of the intervertebral disc at L3/4, and the supraspinous and interspinous ligaments were separated with scissors. Furthermore, after all bilateral intervertebral joints were removed (Fig. 1). We report the degree of instability of the damaged FSU at the results of our past data; the degree of anterior–posterior flexion, left–right bending and rotation in the damaged FSU were approximately 1.5 times to twice larger than those of the intact FSU (Mogi et al. 2007; Oi et al. 2009). Finally, the PS was fixated. We have assumed that the models were done the operations of decompression and instrumentation for degenerative lumbar spine. The size of pedicle screw was φ3.0 × 25 mm and diameter of rod was 5 mm. The name of system was the modified design of Texas Scottish Rite Hospital system, the downsized screws and rods were made of stainless steel. Pedicle screw was inserted while looking at pedicle.

**Fig. 2:**
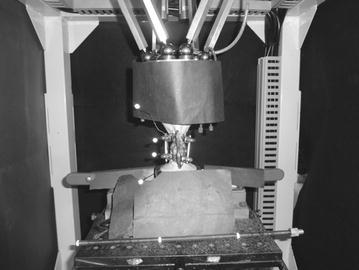
A biomechanical measurement device developed by our laboratory

**Fig. 3:**
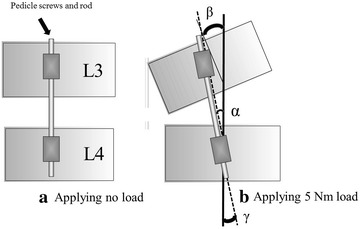
Micromovement in the left anterior flexion. α, Rotational angle of L3 pedicle screw (°); β, rotational angle of L3 vertebral body (°); γ, rotational angle of L4 pedicle screw (°); Rotational angle of L4 vertebral body = 0°; Formula: micromovement of L3 = |α − β|, micromovement of L4 = |γ − 0|

A biomechanical measurement device, a 6-axis material tester to measure spinal strength, developed by our laboratory (Fig. 2), was used (Fujiwara et al. 2006; Kasai et al. 2010); and torque of −5 to 5 Nm was applied at a crosshead angular speed of 0.1°/s. In order to observe the behavior of the screw, markings were made on the screws where the spherical reflective material was attached at the tip of the wire which diameter was 1 mm and was strong enough to exclude its own movement. Then, wires were inserted in the left side of the L3 and L4 vertebrae; two digital video cameras were set so that the imaging direction became ±45° to the vertebral body insertion direction of the left side PS, and bend test movies in the left anterior flexion direction and right posterior flexion direction were taken. By automatically following each marker on the movie obtained by the above method using three-dimensional movie measurement software (Move-tr/3D; Library Co., Ltd., Tokyo, Japan), screw rotational angles at L3 and L4 were measured. As for the rotational angle of the vertebral body, it was determined to be the value calculated from the torque head of the tester for the L3 vertebral body, whereas it was determined to be 0° for the L4 vertebral body, because it was the fixed end. Then, the rotational angles of the vertebral body and the screw at the time of applying a ±5 Nm load in each direction were calculated based on those at the time of applying no load. The absolute value of the difference in the rotational angles between each the inserted screw and vertebral body at L3 and L4 in the left anterior flexion and right posterior flexion was defined as rotational micromovement (Fig. 3). The specimen loaded and rotated three times, and then, we have adopted the third data. A Mann–Whitney U test was used to test differences between two related groups, and p < 0.05 was taken as a significant difference.

The animal experiments in this paper comply with the Principles of Laboratory Animal Care (NIH publication No. 85–23, revised 1985), the OPRR Public Health Service Policy on the Humane Care and Use of Laboratory Animals (revised 1986) and the U.S. Animal Welfare Act, as amended, were followed, as well as specific national laws. And, this study was performed with the approval (No. 1449) of the ethics committee of our university.

## Results

In the left anterior flexion direction, the mean rotational angle of the L3 vertebral body was 2.1° ± 0.5° (mean ± SD), the mean rotational angle of the screw inserted into L3 was 0.9° ± 0.3°, the mean rotational angle of the L4 vertebral body was 0° because it was defined as the fixed end, and the mean rotational angle of the screw inserted into L4 was 0.5° ± 0.3°. These results showed that there was a significant difference (p < 0.05) in the rotational angle between the screw and the vertebral body for both the L3 and L4 vertebral bodies, and mean rotational micromovement was 1.2° ± 0.5° at L3 and 0.5° ± 0.3° at L4.

In the right posterior flexion direction, the mean rotational angle of the L3 vertebral body was 1.8° ± 0.6°, the mean rotational angle of the screw inserted into L3 was 0.9° ± 0.2°, the rotational angle of the L4 vertebral body was 0° because it was defined as the fixed end, and the mean rotational angle of the screw inserted into L4 was 0.7° ± 0.2°. These results showed that there was a significant difference (p < 0.05) in the rotational angle between the screw and the vertebral body in both the L3 and L4 vertebral bodies, and mean rotational micromovement was 0.9° ± 0.4° at L3 and 0.7° ± 0.2° at L4.

## Discussion

Pedicle screw loosening is one of the most frequently reported complication of spinal fixation. Ohtori et al. (2013) reported 15 (14.7 %) loosened pedicle screws in a total of 102 patients with osteoporosis. The screw loosening is usually a consequence of pseudoarthrosis and may be occasionally associated with screw breakage and progressive kyphosis (Berjano et al. 2013; McLain et al. 1993). It is also known that this screw loosening often occurs in patients with multilevel fusion (Schatzker et al. 1975) besides osteoporotic patients.

However, the cause to loosening pedicle screws has not been sufficiently clarified (Mehmanparast et al. 2014). As for the loosening of the screw, Inceoğlu et al. (2008) and Costa et al. (2013) reported that various factors, such as the diameter and material of the screw, or the material and angle of the bone, are involved. Law et al. (1993) and Okuyama et al. (2000) reported that cyclic caudocephalad toggling caused by the craniocaudal screw may be the cause of loosening. We have investigated the biomechanical study and produced the results of weakness of PS fixation under rotational stress, and it may be the cause of loosening of the screw. In our results, there were significant differences (p < 0.05) in the rotational angles between the screw and the vertebral body for both the L3 and L4 vertebral bodies. As far as we have been able to determine, no biomechanical study has been conducted from the viewpoint of rotational micromovement between the screw and the vertebral body, and this paper may be the first study to confirm that there is rotational micromovement between the vertebral body and the screw. This showed the possibility that occurrence of this rotational micromovement caused repeated friction between the bones and the screw within the living body, which led to loosening of the screw or pullout of PS fixation. For this problem, we suggest some ideas. First, expandable screw are useful, because the screws expand in vertebral body and have large contact area to bone. It is under experiment in our laboratory. Additional fixation like sublaminar wirings and trial of raising bone density are also useful.

In this experiment, since it was difficult to obtain the spines of the human cadavers, the spines of boar cadavers were used instead. The advantages on the use of cadaveric boar spines are that the resource of destructive animals can be utilized effectively and that the spines can be obtained easily at very low price. The disadvantage is that the anatomy of the vertebral body of boar spines is significantly different to those of humans. Thus, the results should be interpreted as a proportion or trend rather than quantitatively as angles or ROM (Wasinpongwanich et al. 2014). We would like to perform the same experiment using the spines of human cadavers and conduct a detailed study on rotational micromovement between the pedicle screw and vertebral body by attempting to create a model using the finite element method in the future. The rotational micromovement introduced in this study should be considered in the development and study of new spine instrumentation in the future.

## Conclusion

The behavior of the PS and the vertebral body was biomechanically observed using a PS fixation model of the lumbar spines of boar cadavers. The results showed that rotational micromovement occurred between the PS and the vertebral body, and repeated rotational micromovement might cause loosening of the screw or pullout of PS fixation.

## References

[CR1] Aghayev E, Zullig N, Diel P, Dietrich D (2014). Development and validation of a quantitative method to assess pedicle screw loosening in posterior spine instrumentation on plain radiographs. Eur Spine J.

[CR2] Berjano P, Bassani R, Casero G (2013). Failures and revisions in surgery for sagittal imbalance: analysis of factors influencing failure. Eur Spine J.

[CR3] Costa F, Villa T, Anasetti F (2013). Primary stability of pedicle screws depends on the screw positioning and alignment. Spine J.

[CR4] Fujiwara M, Masuda T, Inaba T (2006). Development of 6-axis material tester for measuring mechanical spine properties. J Robot Mech.

[CR5] Galbusera F, Volkheimer D, Reitmaier S (2015). Pedicle screw loosening: a clinically relevant complication?. Eur Spine J.

[CR6] Inceoğlu S, Kilinçer C, McLain RF (2008). Screw design alters the effects of stress relaxation on pullout. Biomed Mater Eng.

[CR7] Kasai Y, Inaba T, Kato T (2010). Biomechanical study of the lumbar spine using a unilateral pedicle screw fixation system. J Clin Neurosci.

[CR8] Law M, Tencer AF, Anderson PA (1993). Caudocephalad loading of pedicle screws: mechanisms of loosening and methods of augmentation. Spine.

[CR9] McLain RF, Sparling E, Benson DR (1993). Early failure of short-segment pedicle instrumentation for thoracolumbar fractures. A preliminary report. J Bone Joint Surg Am.

[CR10] Mehmanparast HN, Mac-Thiong JM, Petit Y (2014). Biomechanical evaluation of pedicle screw loosening mechanism using synthetic bone surrogate of various densities. Conf Proc IEEE Eng Med Biol Soc.

[CR11] Mogi M, Inaba T, Kasai Y (2007). Influence of injuries of each stabilized element on functional spinal unit. Japanese Journal of Clinical Biomechanics.

[CR12] Ohtori S, Inoue G, Orita S (2013). Comparison of teriparatide and bisphosphonate treatment to reduce pedicle screw loosening after lumbar spinal fusion surgery in postmenopausal women with osteoporosis from a bone quality perspective. Spine.

[CR13] Oi S, Inaba T, Kasai Y (2009). Influence of injuries of each stabilized elements on functional spinal unit under axial rotation. Jpn J Clin Biomech.

[CR14] Okuyama K, Abe E, Suzuki T (2000). Can insertional torque predict screw loosening and related failures? An in vivo study of pedicle screw fixation augmenting posterior lumbar interbody fusion. Spine.

[CR15] Sandén B, Olerud C, Petrén-Mallmin M (2004). The significance of radiolucent zones surrounding pedicle screws. Definition of screw loosening in spinal instrumentation. J Bone Joint Surg Br.

[CR16] Schatzker J, Horne JG, Sumner-Smith G (1975). The effect of movement on the holding power of screws in bone. Clin Orthop Relat Res.

[CR17] Tokuhashi Y, Matsuzaki H, Oda H (2008). Clinical course and significance of the clear zone around the pedicle screws in the lumbar degenerative disease. Spine.

[CR18] Wasinpongwanich K, Sakakibara T, Yoshikawa T (2014). Are deer and boar spines a valid biomechanical model for human spines?. J Spine.

